# Human-Centric Modeling in Metastatic Breast Cancer: Organoids, Organ-on-Chip Systems, and New Approach Methodologies in the Post-FDA Modernization Act 2.0 Era

**DOI:** 10.3390/cancers18071166

**Published:** 2026-04-04

**Authors:** Hissah Alatawi, Haritha H. Nair, Asif Raza, Emiliana Velez, Arun K. Sharma, Satya Narayan

**Affiliations:** 1 Department of Physiology and Aging, College of Medicine, University of Florida, Gainesville, FL 32610, USA; h.alatawi@ufl.edu (H.A.); haritha.nair@neurosurgery.ufl.edu (H.H.N.); emiliana.velez@ufl.edu (E.V.); 2 Department of Molecular and Precision Medicine, Penn State Cancer Institute, Penn State College of Medicine, Hershey, PA 17033, USA; mraza@pennstatehealth.psu.edu (A.R.); asharma1@pennstatehealth.psu.edu (A.K.S.)

**Keywords:** metastatic breast cancer, patient-derived organoids, organ-on-a-chip, tumor microenvironment, new approach methodologies (NAMs), drug resistance, spatial multi-omics, human-centric oncology, precision medicine, AI-driven diagnostics

## Abstract

Metastatic breast cancer (MBC) remains an overwhelming clinical challenge due to its inherent clonal evolution and the frequent development of drug resistance. A significant hurdle in therapeutic discovery is the reliance on traditional 2D cell cultures and animal models, which often fail to accurately replicate human tumor pathophysiology or predict clinical responses. Consequently, the field of oncology is increasingly exploring a transition towards human-centric research that prioritizes biological data derived directly from patients. Considering the FDA Modernization Act 2.0 and the 2025 FDA Roadmap, frameworks are being established to explore the integration of new approach methodologies (NAMs)—including patient-derived organoids (PDOs) and organ-on-a-chip (OoC) systems—into the drug development pipeline. This review examines how these platforms aim to better simulate the human physiological environment by capturing the complex architecture and microenvironment of the tumor. We further discuss how the integration of these models with Artificial Intelligence (AI), spatial multi-omics, and real-time liquid biopsies is being investigated to enhance the speed and precision of therapeutic testing. While still in the translational phase, emerging evidence suggests that human-centric platforms may eventually support rapid functional drug screening, potentially informing patient treatment responses within clinically relevant timeframes. Strengthening the biological link between the patient and their longitudinal data represents a promising strategy to address the complexities of MBC and improve clinical outcomes. These human-centric platforms preserve patient-specific tumor heterogeneity, recapitulate microenvironmental interactions, and enable functional drug testing under physiologically relevant conditions, thereby improving translational accuracy compared to conventional models.

## Simple Summary

Traditional preclinical models often struggle to replicate the intricate complexity of metastatic breast cancer, which contributes to high drug-resistance rates and frequent failures in clinical trials. Consequently, regulatory frameworks are increasingly evolving to recognize the potential of human-centric technologies—such as organ-on-a-chip (OoC) systems and patient-derived organoids (PDOs)—to more accurately reflect human biology. Emerging research suggests that integrating these advanced models with Artificial Intelligence (AI) shows significant promise for accelerating drug-sensitivity screening. While these platforms are currently being explored as proof-of-concept tools, they may eventually support more rapid, personalized treatment predictions. Such advancements represent a future direction that could improve clinical outcomes by aligning therapeutic strategies more closely with individual patient data.

## 1. Introduction

One of the most significant regulatory transformations since the 1938 Federal Food, Drug, and Cosmetic Act is now underway. With the passage of the FDA Modernization Act 2.0 and the subsequent release of the 2025 FDA Roadmap, the United States has reduced the regulatory requirement for routine animal testing and enabled alternative approaches. These landmarks authorize the use of new approach methodologies (NAMs)—including patient-derived organoids (PDOs), organ-on-a-chip (OoC) systems, and AI-driven computational models—to provide evidence of safety and efficacy for investigational new drug (IND) applications. This shift is particularly critical for diseases like metastatic breast cancer (MBC), where traditional animal research has frequently failed to translate into clinical success. MBC represents a particularly relevant disease context for the implementation of these regulatory advances due to its pronounced biological heterogeneity, dynamic clonal evolution, and organ-specific metastatic tropism [1,2,3,4]. Owing to therapeutic pressure, MBC undergoes continuous adaptation, creating spatial and temporal divergence between primary and metastatic lesions [5,6]. Conventional preclinical models, particularly static 2D cultures and murine systems, fail to capture these evolving dynamics, including tumor microenvironment interactions and site-specific metastatic niches. Thus, human-centric platforms such as PDOs and OoC systems are uniquely positioned to address these limitations by preserving patient-specific heterogeneity and enabling functional assessment under conditions that more closely reflect metastatic progression. By prioritizing human-centric biological data, these new regulations provide a clear path to bridge the translational gap and accelerate the delivery of effective therapies to patients.

### 1.1. Pathophysiological Complexity of Metastatic Breast Cancer (MBC)

MBC represents an aggressive and multifaceted clinical scenario, characterized by significant biological challenges and often limited success in durable treatment [7,8]. Globally, breast cancer remains the most prevalent malignancy among women and a primary driver of cancer-associated mortality [1]. A particularly challenging aspect is the frequent metastasis of breast cancer to distant physiological niches, such as the bone, lungs, liver, and brain [9]. These metastatic lesions often exhibit poor responsiveness to conventional chemotherapy compared to primary tumors, rendering standard treatments ineffective in advanced stages [10,11,12,13]. The genetic heterogeneity and variable clinical behavior of the disease further complicate the identification of effective therapeutic regimens [2,7]. The inherent aggressiveness of MBC, coupled with systemic disparities in therapeutic response, underscores a critical gap in current modeling strategies and necessitates the development of platforms that can accurately predict the metastatic cascade.

Breast cancer pathology is characterized by significant histological and molecular heterogeneity, encompassing distinct subtypes defined by hormone receptor status (ER, PR) and HER2 expression, as well as triple-negative phenotypes with aggressive clinical behaviour [1,2]. In the metastatic setting, tumor cells acquire additional alterations that facilitate invasion, immune evasion, and colonization of distant organs such as bone, lung, liver, and brain [2,9]. These pathological features contribute to therapeutic resistance and underscore the need for models that accurately capture disease complexity.

### 1.2. Limitations of Conventional Two-Dimensional (2D) Cell Cultures and Animal Models

For several decades, preclinical breast cancer research has relied heavily on conventional 2D cell cultures and murine models. While 2D systems have facilitated early-stage drug discovery, they possess substantial limitations, including a lack of stromal cell populations, the absence of three-dimensional (3D) spatial architecture, and an inability to represent inter- and intra-tumor heterogeneity [10,13,14]. Cells cultured in 2D environments undergo morphological changes and cytoskeletal rearrangements, leading to artificial polarity and aberrant gene expression that limit their translational power [15]. Furthermore, these models cannot simulate the physiological gradients of oxygen, nutrients, and drug penetration characteristic of in vivo tumors [16].

Animal models, predominantly murine xenografts, have been widely utilized to investigate tumor biology; however, they present significant species-specific drawbacks. Disparities in physiology, immune system regulation, and metabolic responses to anti-cancer agents often lead to discrepancies between animal findings and clinical outcomes [7,17,18]. Notably, the concordance rate between animal safety data and clinical trial success is approximately 9%, with most drugs failing in human trials despite encouraging preclinical results derived from animal data [19,20]. While patient-derived xenografts (PDXs) offer relative improvements, they are resource-intensive and raise ethical concerns [21,22,23,24]. This translational bottleneck emphasizes the urgent need for human-centric in vitro models that more faithfully replicate the human TME and systemic interactions.

### 1.3. Regulatory Drivers and the 3Rs Framework

**Figure 1:**
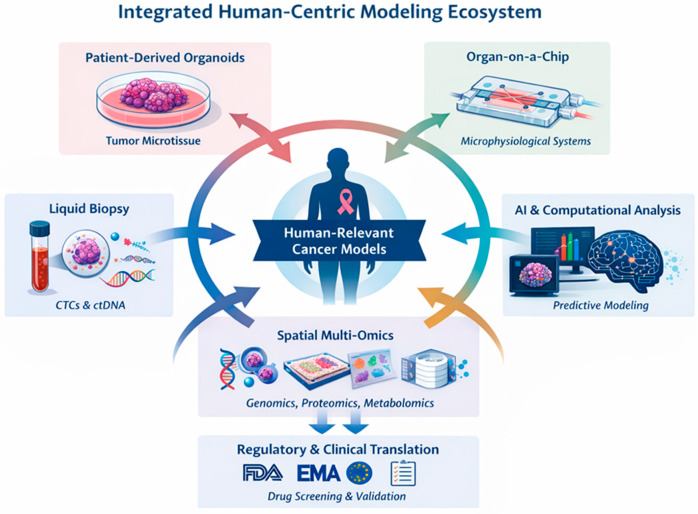
3Rs-Driven Regulatory and Technological Transition Toward Human-Centric Cancer Modeling. Schematic overview of the evolving preclinical ecosystem guided by the 3Rs principles—Replacement, Reduction, and Refinement—and supported by regulatory initiatives promoting scientifically validated non-animal alternatives. Patient-derived organoids, organ-on-a-chip microphysiological systems, and liquid biopsy platforms are integrated with spatial multi-omics and AI-based computational modeling. Together, these interconnected technologies form a unified human-centric framework that enhances translational relevance and accelerates drug discovery while reducing reliance on animal models.

The recognition of these limitations has spurred a global transition toward non-animal alternatives, underpinned by the 3Rs principles—Replacement, Reduction, and Refinement. These efforts are supported by legislative directives such as the European Commission’s Directive 2010/63/EU, which aims to phase out animal testing as scientifically valid alternatives become available [13,25,26,27,28]. The development of advanced non-animal models is driven by the imperative to represent the characteristic heterogeneity of human breast cancer while reducing the scientific burden of animal use [13,29]. This transition is a strategic priority for improving scientific rigor and the efficiency of drug discovery, moving toward a more human-centric approach in preclinical research [30]. Collectively, these technological and regulatory shifts support a transition towards an integrated human-centric modeling ecosystem in which patient-derived platforms, advanced microphysiological systems, real-time molecular monitoring, and computational analytics converge within a unified translational framework (Figure 1) [31]. Moreover, this regulatory shift is particularly relevant in MBC, where preclinical models have demonstrated limited predictive validity for clinical outcomes [19,20,32]. High rates of therapeutic failure in late-stage clinical trials reflect the inability of conventional systems to model metastatic dissemination, immune evasion, and organ-specific microenvironmental adaptation. Human-relevant NAMs directly address these gaps by enabling patient-specific modeling of metastatic niches and therapeutic response, thereby aligning regulatory innovation with disease-specific translational needs.

To facilitate interpretation of the current evidence landscape, we provide a summary framework categorizing representative studies by platform type, disease context, sample source, analytical endpoint, evidence level, and key translational limitation (Table 1).

## 2. Architecture and Organotypic Culture Systems

### 2.1. Biomimetic Advantages of 3D over 2D Systems

Unlike traditional 2D monolayers, 3D systems promote fundamental cell–cell and cell–extracellular matrix (ECM) interactions [15,57,58]. Cell culture models represent an advancement because they effectively mimic the complex architecture of primary tumors [15,59,60]. This architectural fidelity allows for the recapitulation of key in vivo tumor characteristics, including cellular heterogeneity, hypoxic regions, and more accurate gene expression patterns [15,61,62]. Furthermore, 3D cultures maintain the natural morphology and polarity of tumor cells and generate physiological concentration gradients of oxygen and nutrients, which fundamentally alter cellular behavior, leading to more relevant drug responses. For instance, 3D breast cancer cultures have demonstrated decreased proliferative rates and increased resistance to drugs such as cisplatin compared to 2D models, largely due to TME-mediated interactions (Table 2). Not all 3D systems offer equivalent biological fidelity; while spheroids provide simplicity and scalability for high-throughput screening, but they lack structural complexity and stromal integration [12,14,15]. Organotypic cultures improve extracellular matrix interactions and signaling fidelity but remain limited in long-term heterogeneity preservation [14,15,63]. In contrast, patient-derived organoids retain genetic and phenotypic characteristics of the original tumor, including intratumoral heterogeneity [3,21,39,64], while lacking full vascularization and immune components [21,55]. These distinctions are particularly relevant in metastatic modeling, where processes such as invasion, intravasation, and organ-specific colonization require systems that capture both structural complexity and multicellular interactions. Thus, model selection must be aligned with the specific biological question being addressed rather than models being viewed as interchangeable platforms.

### 2.2. Spheroids and Heterotypic Microenvironments

Within 3D modeling, various platforms provide increasing levels of complexity. Spheroids are among the simplest 3D models, consisting of spherical aggregates that self-assemble or are forced to aggregate using low-adhesion techniques [12,14,15,30,70,71]. Spheroids exceeding 500 µm in diameter often exhibit properties akin to avascular tumors, including pathophysiological gradients that form a physical barrier to drug transport [30,72]. Organotypic cultures involve the growth of cancer cells within a semi-solid ECM, which effectively recapitulates growth kinetics and signaling pathway activity [14,15,63,73]. These models are particularly suitable for high-throughput screening (HTS) assays [15,74].

A crucial advancement is the development of heterotypic models, which incorporate multiple cell types to recapitulate the intricate interactions within the TME [15,75]. Breast cancer cells are frequently co-cultured with cancer-associated fibroblasts (CAFs) and immune components like Natural Killer (NK) cells [16,76]. For example, the presence of CAFs can alter the expression of molecules like MICA/B and PD-L1, while also promoting tumor cell aggregation [16,40,77]. This evolution from homotypic to complex heterotypic models is vital, as these interactions enable tumor cells to escape immune surveillance and drive therapeutic resistance [5,16,78].

### 2.3. AI-Driven Organoid Assessment and Automation

Although breast cancer organoid systems have rapidly increased in complexity, measurement strategies have often lagged. Many laboratories still rely heavily on manual microscopic judgment, simple viability endpoints, and a limited number of readouts that fail to fully capture the inherent heterogeneity of 3D systems. These methodological limitations directly impact the reliability of drug response predictions. Variability in imaging conditions, operator-dependent scoring, and inconsistent endpoint selection can introduce significant experimental noise, reducing reproducibility across experiments and laboratories [6]. In the context of translational applications, such variability may lead to inconsistent classification of drug sensitivity or resistance, ultimately limiting the predictive value of organoid-based assays. Furthermore, the development of standardized, quantitative, and reproducible readouts is not merely a technical improvement, but a prerequisite for clinical implementation. Therefore, AI-driven image analysis and automation are now becoming more than just additional tools; they are now essential for ensuring reproducibility [79]. This is particularly critical for patient-derived breast cancer models, where AI can consistently quantify morphological features—such as diverse cell populations—that are difficult to score visually [51].

Recent AI-enabled high-content 3D imaging systems illustrate this shift by enabling multi-parametric assessment of organoid morphology and viability patterns at a single-cell or near-single-cell level. These systems utilize deep learning models trained on complex 3D datasets to detect subtle but biologically significant phenotypic changes, such as budding behavior or lumen collapse [53,80]. For breast cancer research, automation serves as an important quality-control factor; automated workflows preserve the fragile structure and metabolic health of patient-derived models more consistently than manual pipetting [6,52]. Furthermore, machine learning frameworks like Mask R-CNN can extract phenotypic fingerprints—including texture and boundary irregularity—which may predict therapeutic response earlier than standard assays [33,81].

Despite these advances, several practical limitations must be considered. AI models are highly dependent on the quality and diversity of training datasets, and insufficient representation of biological variability can introduce bias and limit generalizability [34,53,79]. Differences in imaging platforms, staining protocols, and culture conditions across laboratories may further compromise model transferability. Additionally, the black-box nature of certain deep learning models poses challenges for interpretability, which is particularly important in clinical decision-making contexts. Addressing these issues will require standardized data acquisition protocols, multi-center validation, and the development of explainable AI frameworks.

### 2.4. Translational Barriers and the Gap Between Performance and Clinical Utility

While the integration of AI into breast cancer organoid research offers transformative potential, significant hurdles remain before these platforms can transition from laboratory tools to clinically reliable decision-support systems. Current literature frequently highlights high technical metrics—such as segmentation accuracies exceeding 90% or AUC values above 0.90 [6,53]—yet these figures often fail to reflect the complexities of real-world clinical application.

#### 2.4.1. Dataset Bias and the Risk of Overfitting

A primary constraint in AI-driven organoid assessment is the reliance on relatively small, highly curated datasets for model training. In the context of (MBC), where patient-derived samples are precious and limited in number, models are at high risk of overfitting. A model may achieve exceptional performance by memorizing the specific imaging artifacts, lighting conditions, or morphological quirks of a single laboratory’s repository, but fail entirely when presented with data from a different institution. Furthermore, annotation quality remains a source of significant ground-truth uncertainty; the manual labeling of 3D structures is subjective, and inter-observer variability among pathologists can induce human bias directly into algorithmic architecture.

#### 2.4.2. Internal vs. External Validation and Model Transportability

Most of the current AI frameworks in the field rely heavily on internal validation (e.g., cross-validation within the same dataset). However, external validation—testing the model on entirely independent datasets from different clinical sites—is frequently absent. This leads to the problem of domain shift, where minor differences in organoid culture media, extracellular matrix (ECM) thickness, or imaging hardware (e.g., different confocal versus widefield settings) degrade model performance. For a platform to be transportable, it must demonstrate resilience to these site-specific variables, a level of robustness that has yet to be widely demonstrated in the current literature.

#### 2.4.3. Disconnect Between Image Performance and Clinical Response

Critically, high performance in image-based tasks (such as accurately classifying an organoid as alive or dead) does not automatically translate into clinically reliable response prediction. A model may excel at identifying morphological shifts in a 14-day in vitro window, yet these shifts may not correlate with the complex, multi-year survival outcomes of a metastatic patient. Bridging this gap requires moving beyond static image-based metrics toward longitudinal, multi-omic integration that accounts for systemic factors—such as drug pharmacokinetics and immune pressure—that are not captured in isolated organoid images. Until multi-center prospective validation is achieved, AI-driven assessments should be viewed as complementary biological readouts rather than definitive clinical predictors.

## 3. Patient-Derived Organoids (PDOs): Functional Avatars in Personalized Medicine

Over the past decade, substantial progress has been made in the development of organoid and microphysiological systems as advanced preclinical models. Early work established that patient-derived organoids preserve key histological, genetic, and transcriptomic features of the original tumor, enabling accurate modelling of tumor heterogeneity and drug response [35,36]. Subsequent studies have demonstrated the scalability of organoid biobanks for high-throughput drug screening and biomarker discovery, further supporting their translational potential [4,37]. In parallel, organ-on-a-chip technologies have evolved to incorporate dynamic microfluidic environments that recapitulate tissue–tissue interfaces, vascular perfusion, and biomechanical forces. These systems have been successfully applied to model tumor progression, metastasis, and drug transport under physiologically relevant flow conditions [38,48,82]. More recent advances include multi-organ chip platforms capable of simulating systemic interactions and metastatic spread across organ-specific niches, providing a more integrated representation of human physiology [65,83]. Together, these studies demonstrate that both organoid and OoC platforms have progressed from proof-of-concept systems to increasingly sophisticated models with growing relevance for translational oncology.

### 3.1. Genomic Fidelity and Heterogeneity Recapitulation

Patient-derived organoids (PDOs) represent a significant leap in preclinical modeling, serving as personalized in vitro avatars. Generated directly from surgically resected tissues or biopsies, PDOs preserve the genetic, histological, and phenotypic features of the original tumor [3,21,39]. They retain key clinical biomarkers, such as estrogen receptors (ER), progesterone receptors (PR), and HER2 status, which are essential for targeted therapies [1,18,64]. A paramount advantage of PDOs is their ability to capture the inherent heterogeneity of cancer, including the coexistence of stem-like and differentiated cells, which is critical for studying therapeutic resistance and clonal evolution [21,39,84]. With success rates for breast cancer organoid culture reaching up to 87.5%, they offer a robust and reproducible framework for elucidating cellular interactions [85].

### 3.2. Clinical Correlation and Biobanking Applications

The unique characteristics of PDOs support their use in preclinical drug screening and the establishment of living biobanks [86,87]. Once genetically and epigenetically characterized, these organoids allow for large-scale drug screening to identify compounds targeting specific features of breast cancer [21,55]. A compelling aspect of PDOs is their demonstrated ability to predict clinical outcomes, with high concordance rates between organoid and patient responses [1]. Some studies have reported high concordance rates between PDO drug responses and patient outcomes [4,88,89]. Ongoing prospective clinical trials (e.g., NCT06468124) are currently assessing the capacity of PDOs derived from metastases to predict survival outcomes and treatment sensitivity [56,90,91,92].

Several studies have demonstrated that patient-derived organoids can recapitulate patient-specific therapeutic responses with high concordance, supporting their application as functional precision oncology tools [56,88,93]. For instance, organoids generated from breast cancer biopsies have been shown to predict response to neoadjuvant chemotherapy, with concordance rates exceeding 80% between in vitro drug sensitivity and clinical outcomes [93]. Similarly, longitudinal organoid models derived from metastatic lesions have enabled the identification of evolving resistance mechanisms under therapeutic pressure, providing a dynamic platform for treatment adaptation [3,4]. In addition, recent studies utilizing patient-derived organotypic tumor spheroids (PDOTS) have demonstrated the capacity to evaluate immunotherapy responses within a preserved tumor microenvironment, highlighting their potential for testing immune-based strategies [41,94,95]. Collectively, these studies support the use of organoid-based platforms as clinically relevant functional avatars capable of bridging the gap between preclinical modelling and patient-specific therapeutic decision-making.

### 3.3. Current Constraints and Future Refinement

Despite their advantages, current PDO models face limitations, including the incomplete recapitulation of the full TME. Most models lack functional blood vessels and a complete complement of immune cells, which can hinder the comprehensive modeling of systemic drug responses [4,21,87,96]. Furthermore, challenges in standardization, inconsistent organoid sizes, and relatively long culture periods (exceeding several weeks) impact their immediate clinical utility for urgent diagnostic scenarios [4,97,98]. These limitations highlight the need for further technological integration with microfluidics or bioprinting to create more comprehensive systems.

Despite promising early concordance data and rapid technological refinement, several factors currently limit broad clinical generalization. Inter-laboratory variability in culture conditions, extracellular matrix (ECM) composition, growth factor supplementation, passage conditions, and analytical pipelines can alter organoid phenotype and influence drug response [6,97,98]. As a result, standardization across institutions remains incomplete, and reproducibility across laboratories remains a significant challenge. Large-scale, prospective multi-center validation studies directly comparing organoid- or chip-guided therapeutic decisions with standard clinical care are required to establish standardized protocols and confirm that PDO-based predictions are consistent and clinically actionable across diverse patient populations [55,56,86]. Furthermore, most of these platforms do not yet fully recapitulate systemic pharmacokinetics, endocrine signaling, or whole-body immune dynamics [38,43,48]. Accordingly, current evidence supports the integration of these models as complementary components within translational pipelines rather than definitive replacements for existing preclinical systems [19,32]. The lack of standardized analytical pipelines—specifically regarding thresholding for drug response versus resistance—remains a hurdle; without a consensus on these metrics, the same PDO may yield conflicting clinical recommendations across different institutions.

### 3.4. Integration of Liquid Biopsy with Patient-Derived Organoid (PDO) Models

One of the most transformative trends emerging in 2024–2025 is the increasing intersection of organoid research with real-time patient monitoring, specifically through liquid biopsy approaches. Traditionally, organoids were viewed as static models derived from a single tissue biopsy or surgical specimen and utilized independently of longitudinal clinical follow-up. Currently, the field is increasingly exploring a connected ecosystem in which blood-derived biomarkers inform both organoid derivation and the continuous monitoring of therapeutic response. This integration is particularly compelling in breast cancer, where tumor evolution, emergent resistance, and minimal residual disease (MRD) are dynamic processes that a single, stationary tissue biopsy cannot fully encapsulate.

Liquid biopsy—encompassing circulating tumor DNA (ctDNA) and circulating tumor cells (CTCs)—is becoming increasingly central to breast cancer management. A dual-use strategy is emerging in which CTCs may serve as a potential source for generating patient-matched in vitro models, while ctDNA provides a real-time surveillance signal reflecting tumor burden and molecular evolution. In this framework, a patient’s blood sample may yield both a functional experimental model (the organoid system) and a longitudinal monitoring signal. This creates a closed-loop clinical pathway in which therapies are tested functionally in the organoid model, while the patient’s real-world response is tracked through ctDNA/CTC kinetics [49]. However, such interactions remain investigational and are not yet implemented as continuous or validated clinical workflows.

Despite its conceptual appeal, several barriers currently limit clinical implementation. The frequency and timing of liquid biopsy sampling required for meaningful longitudinal integration remain unclear, and variability in ctDNA shedding across patients can complicate interpretation [49,67]. Furthermore, establishing robust pipelines that link real-time molecular data with functional organoid testing requires substantial logistical coordination and standardized analytical frameworks. Prospective clinical trials validating this integrated approach are still limited, and its impact on clinical decision-making and patient outcomes remains to be fully established.

A large-scale trial—the SURVIVE study (NCT05658172), a Phase 3 randomized controlled trial—is currently evaluating the clinical utility of liquid biopsy-guided follow-up in early-stage survivors to determine whether earlier detection of recurrence can improve overall survival in early breast cancer patients compared with standard care [50]. Another clinical trial, NCT06468124, related to sensitivity of organoids to predict treatment response in extra-cranial metastases is an observational study designed to evaluate how well patient-derived organoids (PDOs) can predict a patient’s response to cancer treatments. However, the transition of these integrated workflows into routine clinical decision-support for MBC is not yet established and remains contingent upon more robust prospective validation. A comparative analysis of advanced human-centric platforms in (MBC) is detailed in Table 2.

#### Translational Barriers to Integrated Monitoring

Despite its conceptual appeal, several barriers currently limit the clinical implementation of liquid biopsy–organoid models. First, the success rates for CTC-derived cultures remain relatively low compared with tissue-based organoids, often due to the limited number of viable tumor cells present in standard blood draws [67]. Second, the logistical turnaround time for establishing, validating, and drug-screening a patient-specific model often exceeds the window required for urgent clinical decision-making. Furthermore, the infrastructure for such sophisticated workflows is currently limited to high-resource academic centers, and the lack of standardized reimbursement and prospective outcome-based validation prevents these dual-tracking strategies from being adopted as a standard of care. Until these logistical and evidentiary gaps are closed, the integration of liquid biopsy and functional organoid testing remains a promising research direction rather than a validated clinical practice.

The translational workflow of patient-derived organoids, from tumor acquisition to clinical decision support and iterative molecular feedback, is summarized schematically in Table 1.

## 4. Organ-on-a-Chip (OoC) and Microphysiological Systems

### 4.1. Microfluidics and Tumor Microenvironment (TME) Biomechanics

OoC technologies represent a new generation of physiological biomimetic systems built upon microfluidic platforms. These systems enable precise control of physicochemical and biomechanical parameters, allowing the recreation of tissue barriers, vascular perfusion, and fluid shear forces in an in vitro setting [43]. By recreating these dynamic elements, OoC models can simulate cancer-on-a-chip environments that maintain tissue-specific functions and realistic drug pharmacokinetics [43,91,99,100,101]. This dynamic control addresses the limitations of non-perfusable 3D models, in which drug delivery and waste removal cannot be properly recapitulated [13,102]. Furthermore, microfluidics provides a platform for the isolation and analysis of extracellular vesicles (EVs), which are primary messengers in TME communications, with improved sensitivity and high throughput compared to conventional isolation methods like ultracentrifugation [103,104].

### 4.2. Recapitulating the Metastatic Cascade

OoC technology is proficient at modeling the multi-step process of metastasis. Various models have successfully reproduced mechanisms such as cellular proliferation, epithelial–mesenchymal transition (EMT), intravasation, and extravasation [44,46,82,105,106]. A significant advancement in this field is the development of multi-OoC systems, which couple various tissue niches with distant organ sites—such as bone, lung, or liver—linked by simulated vascular flow [44,65,66]. These platforms reproduce the dynamic spread of circulating tumor cells (CTCs) to multiple organs, such as lymph vessel-tumor-blood vessel chips (LTB) and lymph node-on-chip (LNoC) systems [44,65]. This integrated approach allows for the study of organ-specific tropism and inter-organ communication, which are central to the progression of metastatic disease and systemic drug effects [47,107,108].

### 4.3. Integrated Efficacy and Toxicity Testing

**Figure 2:**
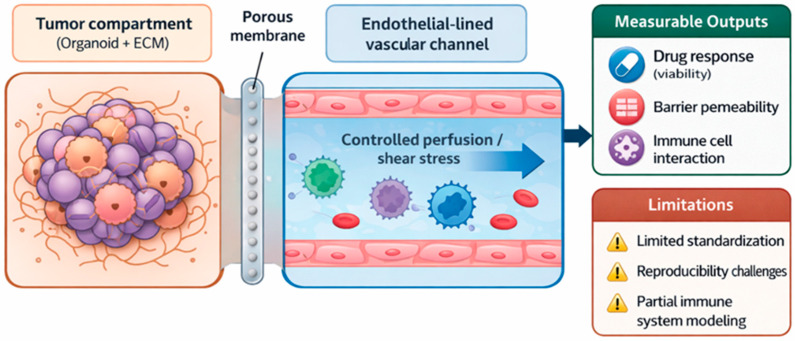
Organ-on-a-chip model for tumor microenvironment simulation. Schematic representation of a tumor compartment interfaced with an endothelial-lined vascular channel under controlled flow conditions. The system enables measurement of drug response, barrier permeability, and immune cell interactions. Current limitations include challenges in standardization, reproducibility, and incomplete modeling of complex tumor–immune dynamics.

Given that concordance rates between animal findings and clinical trials are historically low, OoC systems represent a potential paradigm shift in preclinical testing, reducing late-stage drug failures [32,48,83,109,110]. Tumor-on-chip platforms are increasingly utilized to evaluate the efficacy and toxicity of chemotherapeutic agents, nanomedicines, and nutraceuticals (Figure 2) [44,111,112]. A major advantage of this platform is its ability to assess drug effects on cells collected directly from patients, facilitating personalized testing, and thereby avoiding the species differences in animal models [113,114,115]. Because these systems use human cells, they avoid the species differences in animal models, allowing for a more accurate evaluation of drug toxicity and efficacy [38,43,45].

Despite their advantages, these systems have important limitations, including incomplete representation of systemic physiology, lack of full immune and vascular integration, and challenges in standardization across laboratories [21,38,49,55]. These constraints currently limit their use as standalone clinical decision-making tools and support their role as complementary platforms within translational pipelines.

## 5. Assessing Chemotherapeutic Effects and Metastatic Potential

**Figure 3:**
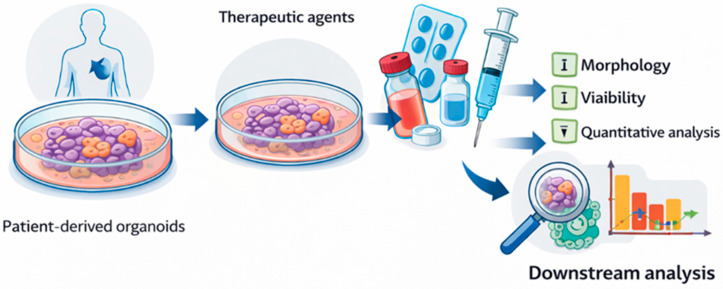
Analytical Assessment of Organoid-based Drug Response. Simplified schematic showing patient-derived organoids exposed to therapeutic agents, followed by evaluation of morphology, viability, and quantitative analysis. This representation reflects commonly used experimental approaches and does not imply a clinically validated workflow.

The multidimensional framework used to quantify chemotherapeutic response across functional, spatial, molecular, and computational domains is illustrated schematically in Figure 3.

### 5.1. Multi-Parametric Readouts for Therapeutic Efficacy

Evaluating drug effects in non-animal models requires precise readouts. While tumor volume and weight are primary outcomes in some studies, cell viability and growth inhibition are more commonly assessed through metabolic activity assays such as AlamarBlue staining [7,116]. Monitoring proliferation markers like Ki67 and p21, together with the induction of apoptosis and senescence, by chemotherapeutic drugs provides a mechanistic insight into reduced cell growth and improved efficacy [15,117,118,119]. Monitoring changes in gene expression and the regulation of microRNAs (miRNAs) helps improve understanding of the emergence of chemoresistance [15,16].

### 5.2. Mechanistic Assays for Dissecting Dissemination

To study the detailed effects of drugs on metastasis, various assays are used to dissect individual steps of the metastatic cascade. Cell migration is fundamentally assessed through wound closure (scratch) assays, which quantify the speed of migration over time [44,120,121]. Transwell and Boyden Chamber assays analyze the ability of single cells to invade through porous membranes coated with matrix proteins [44,122,123]. Additionally, cell exclusion zone assays create voids for cell movement that are imaged over time [124,125]. Microfluidic assays offer a more controlled environment for studying cell motility and allow for the precise control of chemical gradients, making them particularly useful for investigating the complexities of extravasation [44,65,126,127,128].

### 5.3. Multi-Parametric Readouts: Spatial Transcriptomics, Metabolomic Profiling, and Epigenetic Layers

Rather than functioning as independent analytical layers, these multi-omic approaches are increasingly being integrated to generate a unified understanding of tumor behavior. Spatial transcriptomics provides contextual localization of gene expression, metabolomics captures functional biochemical states, and epigenetic profiling reveals regulatory plasticity. When combined, these data sets enable the construction of multidimensional models that link spatial organization with metabolic activity and transcriptional regulation [129,130,131,132,133,134]. Such integrative frameworks are particularly valuable in metastatic breast cancer, where therapeutic response is governed by complex interactions between tumor cells and their microenvironment. The convergence of these data types, particularly when coupled with AI-driven analysis, allows for the identification of composite biomarkers that are more predictive than single-modality readouts [135].

*Spatial Transcriptomics and Micro-regional Architecture.* Spatial transcriptomics is exceptionally powerful because it preserves tissue architecture, which is typically lost in standard single-cell RNA sequencing (scRNA-seq) workflow [42]. While scRNA-seq offers superior cell-type resolution, it disconnects cells from their physical context within tumor–stroma–immune organization. By integrating scRNA-seq with spatial mapping, researchers can identify tumor micro-regions with distinct cellular compositions and functional states. For example, spatial analyses can reveal organized cancer cell clusters partitioned by stromal components, highlighting the contrast between metabolically active cores and immune-interacting peripheries. This micro-regional perspective is critical, as it directly influences the interpretation of immunotherapy resistance, stromal shielding, and invasive patterns. Furthermore, spatial mapping provides a more accurate assessment of immune infiltration, where the specific localization of T cells and macrophages—whether at the tumor boundary or within the interior—correlates with the success or failure of immune-based therapies [129,130,131,132,133]. A significant advantage of spatial methods is their ability to uncover signaling axes selectively enriched at interfaces, which are often diluted in bulk or even in scRNA-seq analysis. For instance, spatial transcriptomics has identified midkine–nucleolin signaling as being selectively upregulated at the tumor–immune interface, creating an immunosuppressive hotspot [54,136]. Additionally, spatially informed computational tools like NicheNet and CellPhoneDB can be applied with greater biological realism when proximity is known, allowing for the inference of ligand–receptor interactions that are physically plausible in real tissue rather than purely theoretical [120,124,125].

*Functional Metabolomics and Metabolic Crosstalk.* Metabolomic profiling adds a crucial layer by reflecting the functional phenotype of the tumor, rather than merely its potential phenotype. While gene expression indicates metabolic capacity, metabolomics reveals the biochemical state and pathway utilization at the time of measurement [137]. This is particularly relevant in breast cancer, where metabolic rewiring drives growth and therapeutic response, often differing significantly even among tumors with similar transcriptomic profiles. Modern platforms, such as isotope-based metabolite libraries (e.g., UCL-MetIsoLib), enable high-resolution profiling of central carbon metabolism and glycolytic pathways in organoids with a depth that is difficult to achieve in vivo [13]. Such approaches support the identification of metabolic vulnerabilities that may be patient-specific [134].

Recent studies have utilized metabolomic subtyping to categorize triple-negative breast cancer (TNBC) into groups with distinct lipid, oxidation, or dysregulation signatures. For example, a ceramide/fatty acid-enriched subtype may exhibit elevated sphingosine-1-phosphate signaling, and targeting these specific pathways has shown promise in organoid-based testing [138,139]. Furthermore, metabolomics highlights metabolic crosstalk in multicellular systems. Co-culture studies demonstrate that stromal interactions can dramatically reshape tumor metabolism, such as shifting glucose consumption and lactate production in ways that are unpredictable from tumor-only models [140]. Spatially resolved metabolomics also reveals nutrient depletion zones (e.g., glucose or tryptophan) at tumor–immune boundaries, which can actively suppress T-cell function and create niches for immune evasion [141].

*Epigenetic Layers and Regulatory Plasticity.* Epigenetic profiling provides a final regulatory layer that is increasingly recognized for its clinical prognostic value. DNA methylation-based analyses can estimate tumor mitotic age, a metric independent of chronological age that correlates with clinical outcomes. Additionally, RNA modifications like m6A are emerging as key regulators of breast cancer progression through the PI3K/Akt and mTOR pathways [142]. Specific epigenetic axes, such as TRIM46–HDAC1-mediated regulation of DNA repair, have been identified as drivers of chemoresistance in organoid contexts. For metastasis, epigenetic mechanisms that downregulate MHC-I presentation in brain metastatic settings are gaining attention and are best studied in organoid–microenvironment co-cultures where the immune context is preserved [143]. Moreover, epigenetic profiling targeting chromatin regulators could provide better insights into the mechanisms contributing to chemoresistance and thereby redefine current therapeutic strategies against MBC [144]. A practical advantage of these systems is the ability to functionally test and reverse epigenetic changes within the same patient-derived model, providing a direct pipeline from mechanistic discovery to therapeutic validation.

## 6. Emerging Technologies and Future Perspectives

### 6.1. 3D Bioprinting and Genomic Engineering

Novel developments in 3D bioprinting enable the precise fabrication of tumor structures that incorporate multiple cell types and compatible biomaterials like hydrogels [145,146]. Researchers have successfully bioprinted breast cancer models with multi-scale vascularization, allowing for the study of immune cell interactions and drug delivery without the use of animal experimentation [113,147,148,149,150]. Simultaneously, CRISPR/Cas9 genome editing has revolutionized the ability to develop accurate disease models and discover genes involved in cancer progression [151,152,153]. This tool allows for the targeted manipulation of resistance genes, providing a high level of precision in dissecting the genetic drivers of metastasis.

### 6.2. Organoid-Based Testing for CAR-T and Next-Generation Immunotherapies

Immunotherapy development for solid tumors is entering a stage where the limitations of conventional 2D screening and standard murine models are increasingly apparent, particularly for engineered cell therapies such as chimeric antigen receptor (CAR)-T cells [154,155]. While CAR-T therapy has achieved landmark success in hematologic malignancies, translation to solid tumors—including breast cancer—remains challenging. This is largely because of the tumor microenvironment (TME), which is physically dense, immunosuppressive, metabolically competitive, and characterized by profound antigen heterogeneity [156]. Even though the CAR-T cell-based therapies against immune-cold MBC are still in its exploratory phase, the strategy of chemoimmunotherapy could be a promising approach to overcome the hurdles in breast cancer immunotherapy [157]. In metastatic breast cancer research, patient-derived organotypic tumor spheroids (PDOTS) provide a superior experimental platform by recapitulating 3D architecture and antigen heterogeneity [41,94,158]. This enables preclinical testing under conditions that more faithfully reflect patient-specific clinical realities than flat cultures.

*Modeling Resistance and Dysfunction.* Recent progress indicates that CAR-T development for solid tumors is advancing from proof-of-concept into early-phase clinical development, with several groups incorporating organoid-adapted testing into their optimization pipelines [159,160,161]. These systems allow researchers to quantify not just cytolytic activity, but also, they enable the longitudinal assessment of T-cell exhaustion, infiltration, persistence, and cytokine secretion patterns within a 3D architecture. Crucially, organoid-based systems reveal suppressive mechanisms that are often invisible in simplified assays. For example, studies using patient-derived organotypic tumor spheroids (PDOTS) have demonstrated that inhibiting TBK1/IKKε can restore CAR-T activity in suppressive environments and mitigate features of T-cell dysfunction [41]. This is a vital insight, as many CAR-T failures in solid tumors result from microenvironment-driven functional shutdown rather than a lack of initial antigen recognition.

*Advanced Engineering and Logic-gated Designs.* Organoid systems are also indispensable for evaluating advanced engineered constructs designed to minimize off-tumor toxicity. Logic-gated designs, such as SynNotch-type IF–THEN circuits, require dual-antigen recognition before full activation. These 3D models provide a more realistic setting to evaluate whether such logic effectively reduces unwanted activation in healthy-tissue analogs while maintaining potent anti-tumor efficacy [159,162]. Beyond T cells, interest is growing in macrophage-based engineering (CAR-M) and Natural Killer (NK)-cell strategies. Organoid models support the evaluation of CAR-M activity within a tumor-like structure and allow for the testing of combinations, such as macrophage engineering paired with checkpoint modulation (e.g., SIRPα pathway interference), to enhance phagocytic function [161]. In breast cancer specifically, expanded NK-cell approaches in organoid contexts have demonstrated dose-dependent killing and have enabled the mechanistic dissection of resistance patterns related to MHC-I expression and inhibitory signaling.

*Clinical and Regulatory Translation.* From a translational perspective, a major advantage of this approach is the capacity for patient-specific immunotherapy testing. A patient-derived organoid can serve as a functional surrogate to determine if a specific CAR-T construct leads to rapid clearance, partial response with clonal escape, or rapid exhaustion [69]. These data can guide rational clinical decisions, such as the selection of co-targets or microenvironment-modifying adjunct therapies. As regulatory agencies increasingly favor New Approach Methodologies (NAMs) and human-relevant data packages, it is expected that organoid-based evidence will play a central role in early-stage immunotherapy development [163,164]. Ultimately, integrating these platforms into the breast cancer research pipeline demonstrates that organoids are no longer limited to chemotherapy screening but have become essential for guiding the next generation of immune engineering strategies.

### 6.3. Advanced Imaging and Artificial Intelligence

The application of advanced imaging techniques, such as contrast-enhanced MRI and PET/CT, is transforming the assessment of these models [165,166,167,168,169]. AI-powered 3D imaging tools can automatically identify tumor volume and assess distances to anatomical features in vitro [170,171,172]. Deep learning models have demonstrated high accuracy in predicting pathological responses to therapy, often exceeding expert assessment [68].

In imaging-based prediction frameworks, deep learning architectures, typically multi-layer CNN or hybrid radiomic neural network models learn spatial intensity gradients, texture distributions, and volumetric heterogeneity patterns directly from raw imaging datasets. In breast cancer applications, reported predictive performance for treatment response has reached sensitivities between 80–92% and specificities ranging from 75–90%, depending on imaging modality and cohort size [68,170,171,172]. When integrated with high-content organoid imaging, these models can correlate dynamic morphological evolution with transcriptomic or metabolomic features, enabling multimodal prediction rather than single-endpoint classification. However, model robustness remains contingent upon standardized acquisition protocols and balanced training datasets to mitigate overfitting and institutional bias.

**Figure 4:**
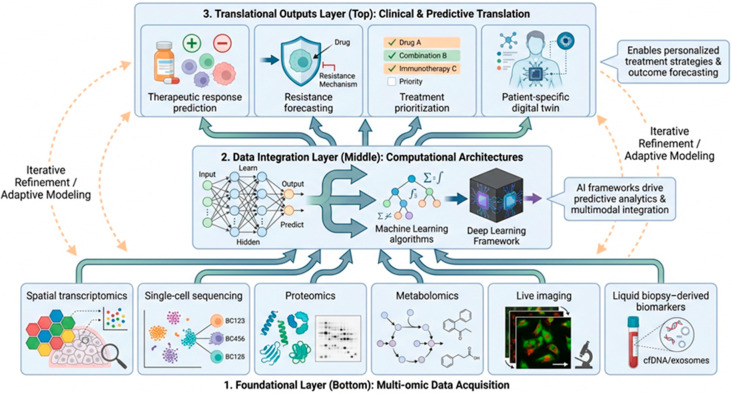
Hierarchical integration of multi-omics data and AI-driven predictive modeling in advanced tumor platforms. Schematic representation of a layered framework integrating multidimensional biological data with computational modeling. The bottom layer depicts multi-omic data acquisition, including spatial transcriptomics, single-cell sequencing, proteomics, metabolomics, live imaging, and liquid biopsy-derived biomarkers. These heterogeneous data streams are integrated within the middle layer using multimodal computational architectures, including machine learning and deep learning frameworks, to enable predictive modeling. The top layer illustrates key translational outputs, including therapeutic response prediction, resistance forecasting, treatment prioritization, and patient-specific digital twin development. Bidirectional feedback loops indicate iterative refinement between experimental platforms and computational models, supporting adaptive and personalized strategies in precision oncology. Dashed connections indicate proposed interactions between experimental platforms and computational models. These relationships are conceptual and do not represent validated or continuously implemented clinical feedback systems.

Furthermore, platforms such as Incucyte, an automated live-cell imaging and analysis system, enable real-time monitoring of growth and apoptosis in response to treatment [173]. The integration of AI and machine learning (ML) optimizes high-throughput screening and drug response prediction, transforming preclinical research into a data-driven science (Figure 4) [4,151,174].

### 6.4. Collaborative Consortia and Standardization

The complexity of breast cancer necessitates shared expertise and standardized protocols. Consortia such as the NCI Cohort Consortium and the Breast Cancer Family Registry (B-CFR) foster collaborative research beyond the scope of individual studies [175,176,177]. Initiatives like the European Commission’s Joint Research Centre (JRC) have cataloged over 900 non-animal models to improve accessibility [178]. This collaborative ecosystem is essential for overcoming scalability limitations and ensuring the widespread adoption and clinical translation of these promising technologies [97].

## 7. Conclusions

The field of MBC research is undergoing a structural and conceptual transformation driven by the limitations of traditional preclinical systems and the emergence of physiologically relevant human-centric platforms [7,18,19,20]. The evidence reviewed herein demonstrates that advanced three-dimensional (3D) cultures, patient-derived organoids (PDOs), and organ-on-a-chip (OoC) microphysiological systems provide superior architectural fidelity, microenvironmental integration, and translational relevance compared with conventional 2D cultures and many animal models [13,30,38,43,91].

Beyond structural mimicry, these systems enable functional interrogation of drug response, metastatic dissemination, immune engagement, and toxicity under conditions that more closely approximate human tumor biology [1,4,44,115]. Importantly, the convergence of organoid biobanking [55,86], spatial and multi-omic profiling technologies [129,130,131,132,133,134], CRISPR/Cas9 genome engineering [151,152,153], 3D bioprinting [145,146], and artificial intelligence-assisted analytics [6,33,53,68,170,171,172] is redefining how therapeutic hypotheses are generated and validated. Rather than relying on reductionist endpoints, these integrated frameworks support multidimensional, data-rich analyses capable of capturing tumor heterogeneity and adaptive resistance mechanisms [2,15,138].

Collectively, these advances signify a decisive transition toward predictive, mechanism-informed, and patient-aligned modelling strategies. By synthesizing current progress in these high-fidelity platforms, this review provides a roadmap for investigating the systemic nature of breast cancer metastasis and identifying vulnerabilities in the metastatic microenvironment. As regulatory landscapes increasingly recognize the scientific and ethical value of New Approach Methodologies [25,28,38], next-generation in vitro systems are poised to improve translational accuracy, reduce late-stage clinical attrition [19,20,32], and accelerate precision oncology for MBC.

## 8. Future Perspectives

The next phase of oncology innovation—and specifically the management of Breast Cancer Metastasis (BCM)—will be characterized by deeper integration, improved standardization, and longitudinal biological connectivity, driven largely by the regulatory flexibility afforded by the FDA Modernization Act 2.0. This landmark legislation, alongside the 2025 FDA Roadmap, has removed the universal requirement for animal testing in drug development, providing a clear mandate for the validation and implementation of new approach methodologies (NAMs). As outlined in this review, these shifts are particularly transformative for BCM, where traditional models have limitations to capture the human-specific complexities of distant site colonization.

First, the integration of structural tumor models with dynamic molecular monitoring will be critical for regulatory qualification. Linking PDO and OoC systems with real-time circulating biomarkers and spatial multi-omic technologies [49,129,130,131,132,133] may enable adaptive experimental designs that mirror tumor evolution. Such approaches are particularly relevant in metastatic disease, where clonal dynamics shift rapidly under therapeutic pressure [2,138], allowing researchers to meet the FDA’s call for more human-relevant longitudinal data. By leveraging the evidence provided here, researchers can better design longitudinal studies that track the transition from dormancy to active metastatic growth.

Second, scalability and reproducibility will determine clinical and regulatory translation. Under the new FDA frameworks, the industry must transition from adapted models to standardized platforms. Harmonized culture conditions, standardized quality-control pipelines, and cross-institutional validation remain necessary to ensure widespread adoption [38,56,178]. Collaborative consortia and shared biobanking infrastructures will be instrumental in establishing the benchmarking frameworks required for these systems to serve as primary evidence in investigational new drug (IND) applications [175,176,177].

Third, computational integration is expected to evolve from simple image segmentation toward predictive modeling of treatment trajectories. AI-driven high-content imaging platforms [6,33,53], combined with radiomics and multimodal analytics [68,170,171,172], may enable probable modeling of therapeutic response. By integrating phenotypic, transcriptomic, and metabolomic layers into unified decision-support tools, these digital twins can complement physical NAMs to fulfill the FDA’s vision of data-driven drug assessment. This review emphasizes that for BCM, these digital twins must specifically account for the unique metabolic and mechanical stresses of the metastatic microenvironment.

Fourth, microphysiological systems are expected to progress toward interconnected multi-organ networks (multi-organ-on-a-Chip) capable of simulating systemic pharmacokinetics, immune interactions, and metastatic niche selection within a single human-relevant framework [38,46,47,179]. These platforms bridge the gap between localized tumor modeling and organism-level assessment, allowing for the simultaneous evaluation of efficacy and off-target toxicity [32,83,109]—a key priority for the FDA in reducing reliance on non-human primates and other animal models. This review serves as a catalyst for these developments by identifying the specific biological parameters necessary to simulate the interaction between breast cancer and various metastatic organs, such as bone, lung, and brain.

Finally, continued regulatory alignment and the ethical emphasis on replacement, reduction, and refinement (3Rs) principles [25,26,27,28] will accelerate the institutional adoption of these technologies. As evidence accumulates demonstrating that these human-centric systems provide superior predictive validity over traditional animal models [19,32], they will transition from complementary tools to foundational components of the drug development pipeline. Within this emerging precision-oncology ecosystem, advanced human-relevant platforms will not merely replace outdated methodologies but will redefine how MBC is modeled, stratified, and therapeutically targeted in an adaptive, data-driven manner.

## Figures and Tables

**Table 1:** Evidence Framework for Human-centric Modeling Platforms in Metastatic Breast Cancer.

Platform	Disease Context	Sample Source	Primary Endpoint	Evidence Level	Translational Limitation
Patient-Derived Organoids (PDOs)	Metastatic breast cancer (MBC); breast cancer (general)	Tumor biopsy (primary or metastatic lesions)	Drug sensitivity, viability, morphological response	Early translational correlation (retrospective studies) [33,34,35,36,37,38]	Limited vascular/immune components; variability in culture conditions; lack of standardized protocols
Organoid Biobanks	Breast cancer (multi-subtype cohorts)	Patient-derived tumor collections	High-throughput drug screening; biomarker discovery	Preclinical + early translational [39,40]	Selection bias; underrepresentation of metastatic niches; scalability challenges
Patient-Derived Organotypic Tumor Spheroids (PDOTS)	Breast cancer; other solid tumors	Fresh tumor tissue (partially dissociated)	Immune response, viability, drug sensitivity	Proof-of-concept (preclinical) [41,42]	Short-term culture; limited expansion; heterogeneity loss over time
Organ-on-a-Chip (Tumor-on-Chip)	Breast cancer; limited MBC-specific studies	Cell lines, PDOs, co-culture systems	Drug response under flow; invasion; barrier function	Preclinical platform development [43,44,45]	Technical complexity; low throughput; reproducibility across laboratories
Multi-organ Chip Systems	General oncology (limited MBC data)	Engineered multi-tissue constructs	Metastatic spread; pharmacokinetics; inter-organ interaction	Proof-of-concept [46,47]	Lack of clinical validation; simplified physiology; scalability
Liquid Biopsy (ctDNA)	MBC (well-studied)	Blood (circulating tumor DNA)	Mutation tracking; minimal residual disease; treatment monitoring	Clinically implemented (selected contexts) [48,49]	Variable shedding; interpretation challenges; not directly functional
Liquid Biopsy (CTCs)	MBC	Blood (circulating tumor cells)	Cellular analysis; potential culture	Proof-of-concept/early translational [49,50]	Low yield; difficulty in expansion; technical variability
AI-based Organoid Analysis	Breast cancer; broader oncology	Imaging data (organoid cultures)	Segmentation, morphology classification, response prediction	Preclinical + early translational [51,52]	Dataset bias; lack of external validation; limited clinical correlation
Spatial Multi-omics Integration	Breast cancer; emerging in MBC	Tumor tissue, organoids	Gene expression, spatial architecture, metabolic profiling	Early translational/exploratory [33,53,54]	High cost; data integration complexity; limited clinical standardization
Integrated PDO + Liquid Biopsy Platforms	Conceptual/emerging MBC studies	Organoids + blood samples	Dynamic therapy adaptation	Hypothesis-generating/emerging [49,55,56]	Logistical complexity; lack of prospective validation; workflow integration challenges

Representative platforms are summarized according to disease context, primary application, level of evidence, and major translational limitations to highlight differences in clinical maturity.

**Table 2:** Comparative Analysis of Advanced Human-Centric Platforms in Metastatic Breast Cancer (MBC).

Platform	Disease Context & Scope	Primary Methodology	Evidence Maturity	Primary Translational Barriers	Key References
Patient-derived organoids (PDOs)	Primary tumor modeling; Metastatic drug-sensitivity screening	3D scaffold-based culture from surgical/biopsy tissue	Level IV: Prospective trials (e.g., NCT06468124)	Culture turnaround time (>3 weeks); Lack of systemic immune/vascular components	[1,21,32,39,56,64]
Organ-on-a-chip (OoC)	Modeling organ-specific tropism (bone, lung, liver)	Microfluidic platforms with physiologic shear & barriers	Level I: Preclinical engineering/foundational	Scaling for high-throughput; Inability to model whole-body pharmacokinetics	[44,47,65,66]
Liquid biopsy-linked models	Longitudinal monitoring; minimal residual disease (MRD)	CTC-derived organoids; ctDNA molecular tracking	Level III: Early translational correlation	Low success rates for CTC expansion; Infrastructure for real-time blood-to-model pipelines	[49,50,67]
AI-enabled analysis	Automated phenotyping; Multi-omic integration	Deep learning (CNNs); Computer vision for 3D morphology	Level II: Validated Biological Readouts	Training dataset bias; Domain shift between labs; Lack of multi-center external validation	[6,33,53,68]
Immune-PDOTS	Modeling CAR-T & immunotherapy resistance	Patient-matched organotypic tumor spheroids	Level II/III: Proof-of-Concept/Correlation	Recapitulating the full immunosuppressive TME; Antigen heterogeneity modeling	[41,69]

## Data Availability

No new data were created or analyzed in this study.

## Abbreviations

The following abbreviations are used in this manuscript:

AI	Artificial intelligence
BCMoC	Breast cancer metastasis-on-chip
BCoC	Breast cancer-on-chip
BCLBoC	Breast cancer liquid biopsy-on-chip
CAF	Cancer-associated fibroblast
Cas9	CRISPR-associated protein 9
CRISPR	Clustered Regularly Interspaced Short Palindromic Repeats
CTC	Circulating tumor cell
ECM	Extracellular matrix
EMT	Epithelial–mesenchymal transition
ER	Estrogen receptor
EV	Extracellular vesicles
HER2	Epidermal growth factor receptor 2
HTS	High-throughput screening
LNoC	Lymph node-on-chip
LTB	Lymph vessel-tumor tissue-blood vessel chips
MBC	Metastatic breast cancer
MDA-MB	MD Anderson-Metastatic Breast
NK	Natural Killer
OoC	Organ-on-a-chip
PDO	Patient-derived organoid
PDX	Patient-derived xenograft
PR	Progesterone receptor
TME	Tumor microenvironment
